# Dataset for evaluating WRF-Chem sensitivity to biogenic emission inventories in a tropical region. Global online model (MEGAN) vs local offline model (BIGA)

**DOI:** 10.1016/j.dib.2021.107438

**Published:** 2021-09-30

**Authors:** F. Cifuentes, C.M. González, B.H. Aristizábal

**Affiliations:** Hydraulic Engineering and Environmental Research Group (GTAIHA), Universidad Nacional de Colombia Sede Manizales, Carrera 27 64-60, Manizales, Colombia

**Keywords:** Tropical Andes, WRF-Chem, Air quality modeling, Ozone, Isoprene, BIGA, MEGAN

## Abstract

This article presents a dataset comparing emissions of Biogenic Volatile Organic Compounds (BVOC) in a zone of complex topography in the tropical Andes, which presents elevations ranging from 250 to more than 4000 m above sea level in a radius of only 50 km. Two approximations were evaluated, (1) online with the Model of Emissions of Gases and Aerosols from Nature (MEGAN) coupled with the Weather Research and Forecast model with Chemistry (WRF-Chem) and (2) offline applying the Biogenic Altitudinal Gradient Model (BIGA). Modeled concentrations of pollutants (mainly isoprene and tropospheric ozone) were obtained with WRF-Chem employing the biogenic emission models mentioned previously. This information identified areas where BVOC emissions vary significantly, comparing the global emission inventory (MEGAN) and the local inventory (BIGA). Re-evaluation of the emission factors and land cover assigned to those areas in the global online biogenic models should be considered in order to reduce the uncertainty in the values. In addition, the dataset shows the impact of the biogenic emission inventories on the air quality simulations on a tropical high mountain area, where vegetation is diverse, and the altitudinal changes influence meteorological variables.

## Graphical abstract

**Figure:**
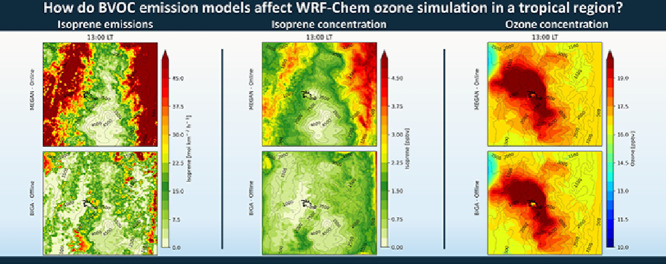

**Specifications Table**

**Table:** 

Subject	Atmospheric Science
Specific subject area	Regional-scale air quality modeling. Biosphere-Atmosphere interactions.
Type of data	Figures Video CSV files GeoTIFF files NetCDF files
How data were acquired	This dataset was generated using the Model of Emissions of Gases and Aerosols from Nature (MEGAN) [1], the Biogenic Altitudinal Gradient Model (BIGA) [2], and the Weather Research and Forecast model with Chemistry Version 3.7 [3].
Data format	Raw Analyzed
Parameters for data collection	- The simulation period was June 3, 00:00 UTC to July 1, 00:00 UTC 2018. - The target domain of the simulations is located in a tropical Andes region in Colombia. The geographic extent is −75.8954, −74.9054, 4.6229, 5.5229. - Spatial and temporal resolution of the emission inventories and simulation outputs is 1 Km – 1 km; 1 h. - WRF-Chem settings were chosen according to the recommendations made by Cifuentes et al. [4].
Description of data collection	- Anthropogenic emissions were retrieved from an existing local emission inventory [5]. - Biogenic emissions were estimated by two approximations: (1) Online using MEGAN [1] coupled with WRF-Chem and (2) Offline using BIGA model [2]. - Thee-dimensional gridded concentrations were simulated using WRF-Chem 3.7. Model settings were defined according to the findings of Cifuentes et al. [4]. - Data were post-processed using NCO operators. - Visualizations were generated using python software.
Data source location	*Institution:* Universidad Nacional de Colombia Sede Manizales. *City/Town/Region:* The geographic extent is −75.8954, −74.9054, 4.6229, 5.5229. The region presents terrain elevations ranging from 250 to more than 4000 m.a.s.l in a radius of only 50 km. *Country:* Colombia.
Data accessibility	Figures and videos are available within this article. The CSV, GeoTIFF, and NetCDF files can be accessed through Mendeley at https://data.mendeley.com/datasets/g8wrxkbzpg/1 or using the https://doi.org/10.17632/g8wrxkbzpg.1

**Value of the Data**

• This dataset is useful to identify areas with major differences in VOC biogenic emissions (BVOC) in the Andes region within a global emission model and a local emission inventory. Results obtained suggest areas that need further study to determine adequate emission factors or land cover classifications to reduce uncertainty in the estimates.
• The dataset provides insights of the influence of BVOC fluxes in atmospheric chemistry of a region of the tropical Andes, through the application of a regional air quality model (WRF-Chem) with the two biogenic emission models (MEGAN and BIGA).
• The dataset can be used to identify areas with critical levels of pollution in the studied area supporting air quality assessment.
• The dataset can be used as a reference for future air quality simulations performed in areas with dense and diverse vegetation, as well as a high climatic variability caused by the complex orography.
• The dataset contains the files needed to elaborate local emission inventories in other regions using BIGA, as well as to run the WRF-Chem simulations.
• A modified version of the WRF-Chem module “module_cbmz_addemiss.F” is included in the dataset. The module was adjusted to include biogenic emissions (isoprene) from the offline biogenic model along with the anthropogenic emission inventory.

## 1 Data Description

The dataset made available through the Mendeley repository (See Specification Table) has three folders. The folder WRF-Chem_Outputs contains two NetCDF files. Each file presents gridded hourly mean values of air quality and meteorological variables specified in Table 1, for the period of analysis (June 3, 00:00 to July 1, 00:00 UTC 2018). In particular, the file CALDAS_WRF_37_MEGAN.nc contains simulated values when using MEGAN model to estimate biogenic emissions, while the file CALDAS_WRF_37_BIGA.nc presents modeled values when the BIGA model is used to supply biogenic emissions.

**Table 1:** Variables included in the WRF-Chem outputs dataset.

Variable name	NetCDF variable	Units	Size
Isoprene emission	E_ISO (BIGA), EBIO_ISO (MEGAN)	mol km^−2^ h^−1^	3D
Isoprene mixing ratio	iso	ppmv	4D
Ozone mixing ratio	o3	ppmv	4D
Carbon monoxide mixing ratio	co	ppmv	4D
Nitic oxide mixing ratio	no	ppmv	4D
Nitrogen dioxide mixing ratio	no2	ppmv	4D
Formaldehyde mixing ratio	hcho	ppmv	4D
Hydroxyl radical mixing ratio	ho	ppmv	4D
Latitude	XLAT	degree north	3D
Longitude	XLONG	degree east	3D
Perturbation pressure	P	Pa	4D
Base state pressure	PB	Pa	4D
x-wind component	U	m s^−1^	4D
y-wind component	V	m s^−1^	4D
z-wind component	W	m s^−1^	4D
Planetary boundary layer height	PBLH	m	3D
Base state temperature	T00	K	1D
Perturbation potential temperature	T	K	4D
Downward short-wave flux at ground surface	SWDOWN	W m^−2^	3D
Surface pressure	PSFC	Pa	3D
2 m specific humidity	Q2	Kg Kg^−1^	3D
2 m temperature	T2	K	3D
Perturbation geopotential	PH	m^2^ s^−2^	4D
Base-state geopotential	PHB	m^2^ s^−2^	4D

*3D variables have the following dimensions (Time: 24, south_north: 100, west_east: 110).

**4D variables have the following dimensions (Time: 24, bottom_top_stag: 35, south_north: 100, west_east: 110).

The folder named BIGA_Inputs contains the information needed to replicate the elaboration of the local biogenic emission inventory. This includes a digital elevation model (DEM) of the area in GeoTIFF format, a land cover and use map (LCU) in GeoTIFF format, and several CSV files containing hourly records of temperature and solar radiations for the meteorological stations specified in Table 2. BIGA source code and tutorials can be download at http://idea.manizales.unal.edu.co/gta/ingenieria_hidraulica/BIGA/index.php.

**Table 2:** Meteorological stations used to run BIGA model.

ID	Latitude	Longitude	Height [m]	Temperature	Solar radiation
1	5.2991	−75.0573	1591	Yes	Yes
2	5.1398	−75.4998	929	Yes	
3	5.3904	−75.4866	2078	Yes	Yes
4	5.0519	−75.5350	1940	Yes	Yes
5	5.1585	−75.5171	2053	Yes	Yes
6	5.0484	−75.5186	1790	Yes	Yes
7	5.0714	−75.5243	2226	Yes	Yes
8	4.9791	−75.6878	1232	Yes	
9	5.3009	−75.3495	3110	Yes	Yes
10	4.8808	−75.3571	4450	Yes	
11	5.0649	−75.4772	1960	Yes	
12	5.0562	−75.4923	2179	Yes	Yes
13	5.4455	−75.5599	840	Yes	
14	5.4531	−75.6540	1239	Yes	
15	5.0911	−75.7900	1728	Yes	Yes
16	5.2090	−75.1407	1450	Yes	

The folder WRF-Chem_Inputs contains the namelist.wps and namelist.input files used to run WRF-Chem. In addition, two NetCDF files containing the local anthropogenic and biogenic emission inventories are provided. Note that for simulating using MEGAN, only the file wrfchemi_anthropogenic.nc is used, as biogenic emissions are estimated online by WRF-Chem. On the other hand, when the local biogenic emission inventory is to be used, the anthropogenic and biogenic emission files need to be added. This can be done employing an NCO operator using the following command “ncbo –op_typ=add wrfchemi_anthropogenic.nc wrfchemi_biogenic.nc wrfchemi_total.nc”.

**Fig. 1:**
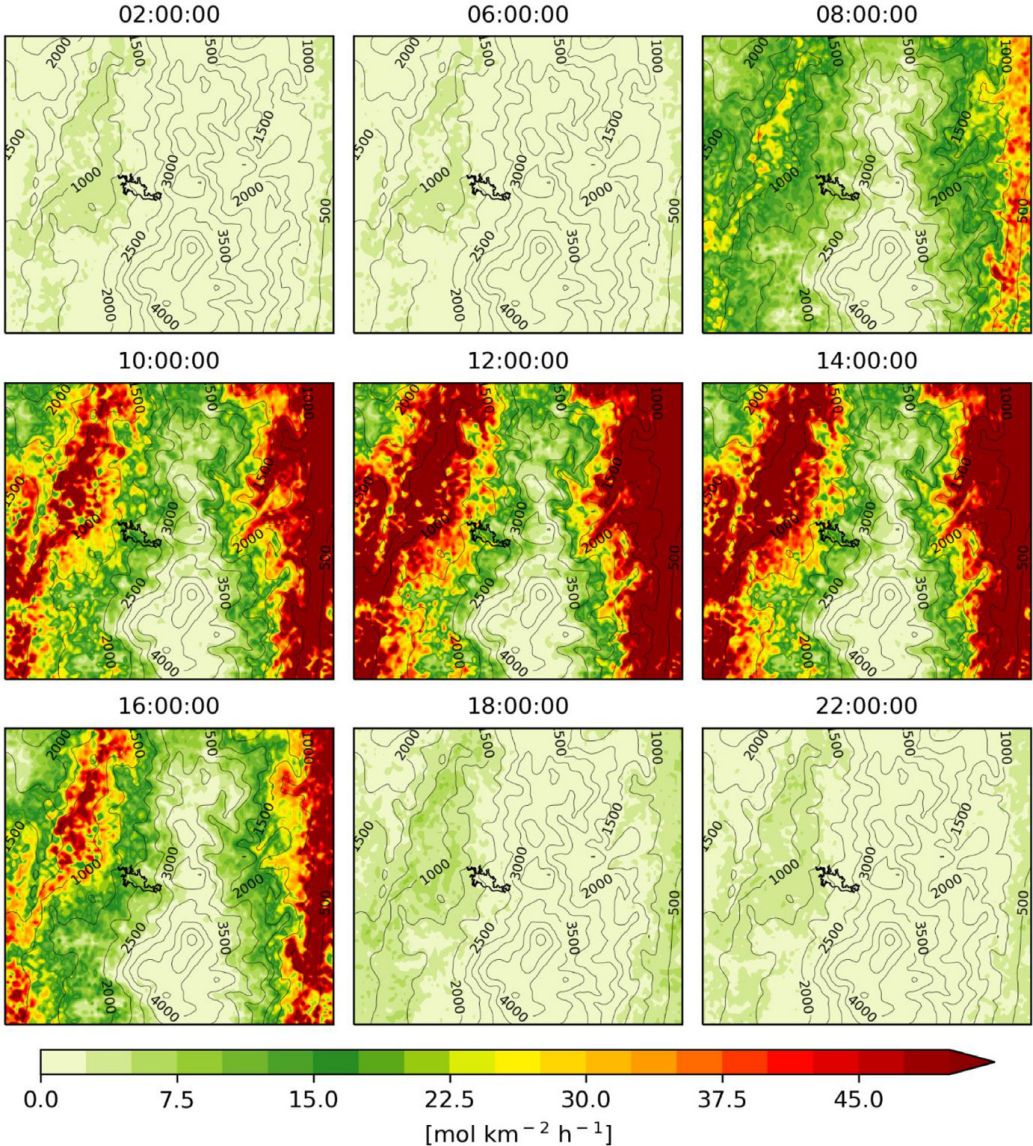
Hourly mean isoprene emissions estimated with MEGAN-WRF-Chem for the period of simulation (June 3, 00:00 UTC to July 1, 00:00 UTC 2018).

**Fig. 2:**
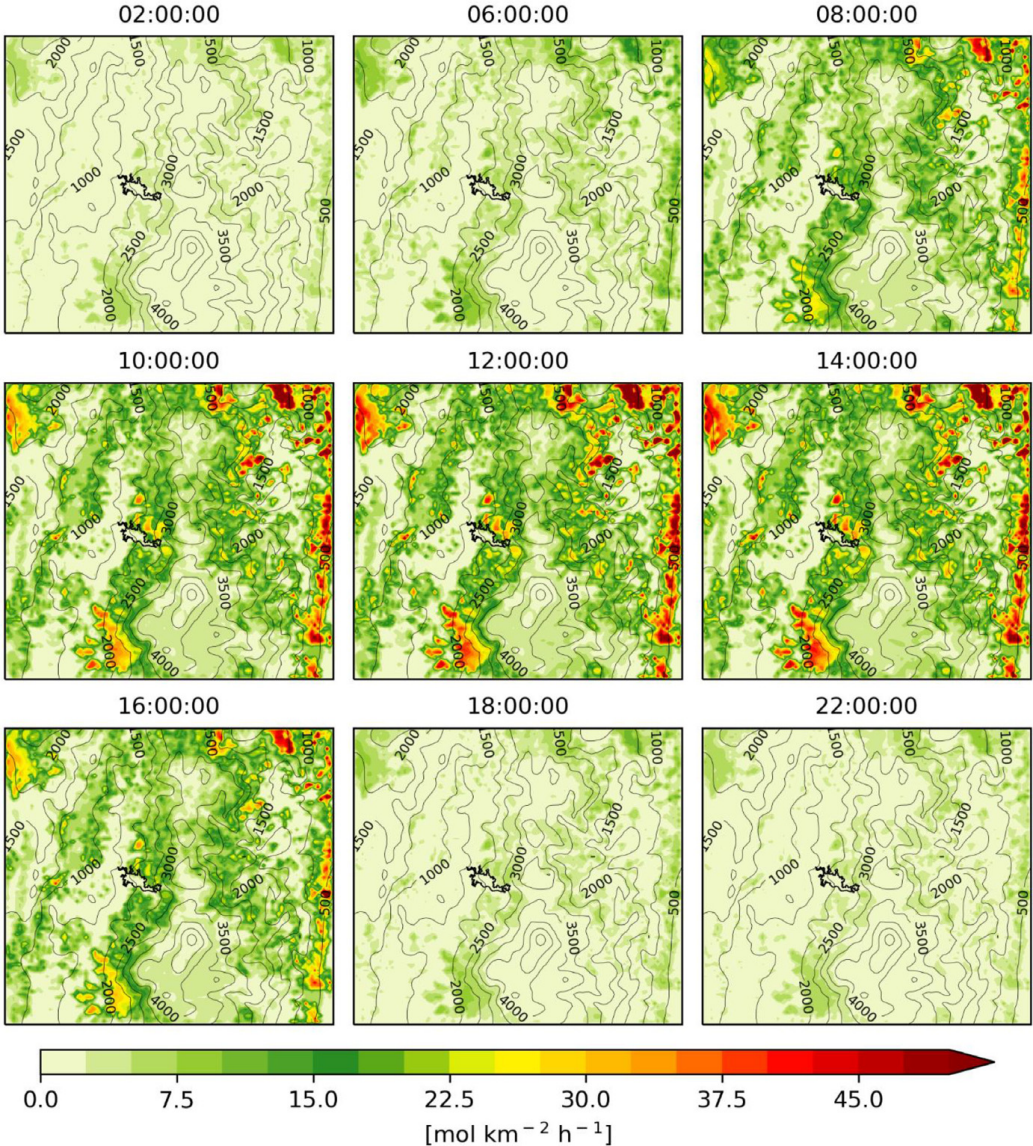
Hourly mean isoprene emissions estimated with BIGA for the period of simulation (June 3, 00:00 UTC to July 1, 00:00 UTC 2018).

**Fig. 3:**
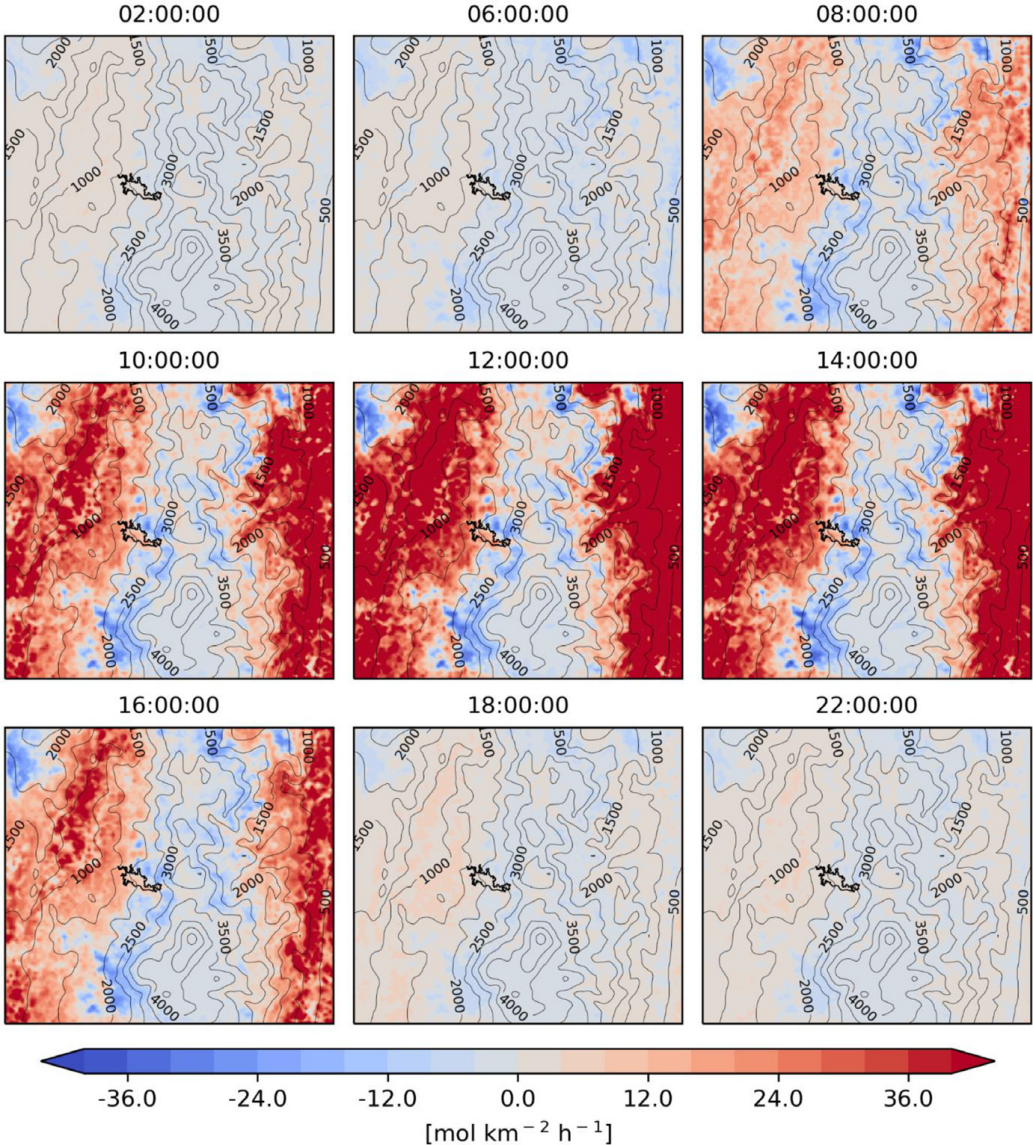
Mean differences of isoprene emissions between MEGAN-WRF-Chem and BIGA (MEGAN minus BIGA) for the period of simulation (June 3, 00:00 UTC to July 1, 00:00 UTC 2018).

**Fig. 4:**
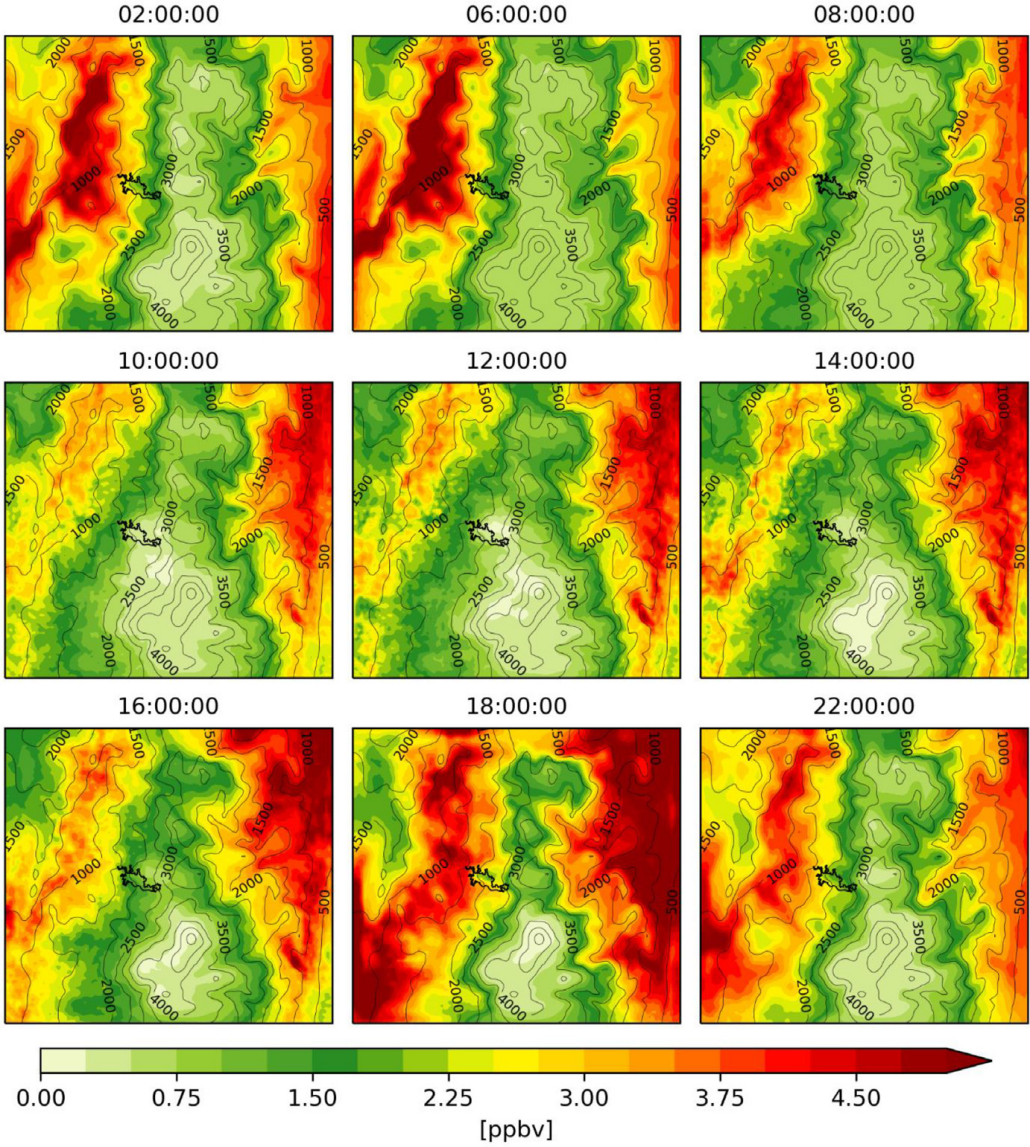
Hourly mean isoprene concentrations estimated with MEGAN-WRF-Chem for the period of simulation (June 3, 00:00 UTC to July 1, 00:00 UTC 2018).

**Fig. 5:**
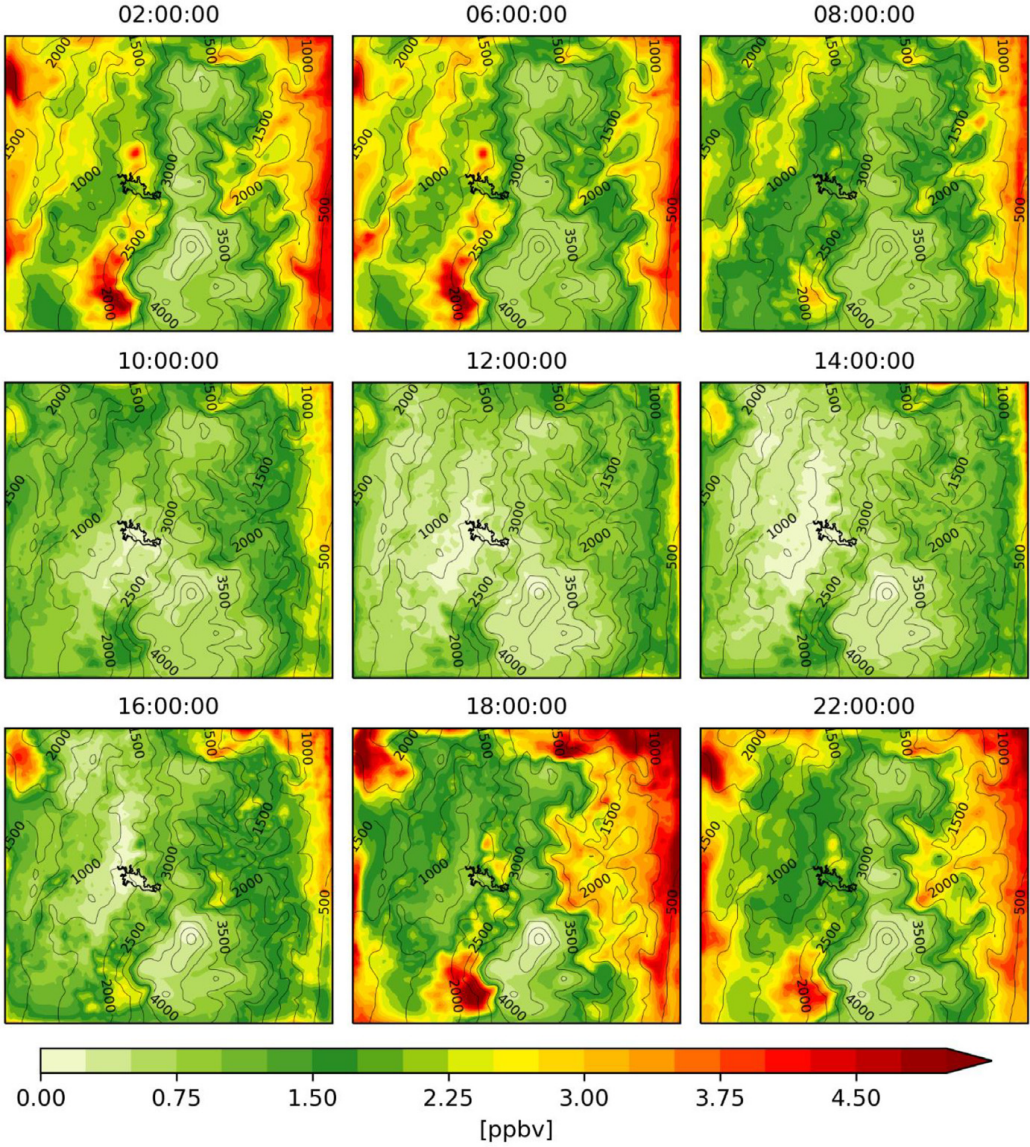
Hourly mean isoprene concentrations estimated with BIGA-WRF-Chem for the period of simulation (June 3, 00:00 UTC to July 1, 00:00 UTC 2018).

**Fig. 6:**
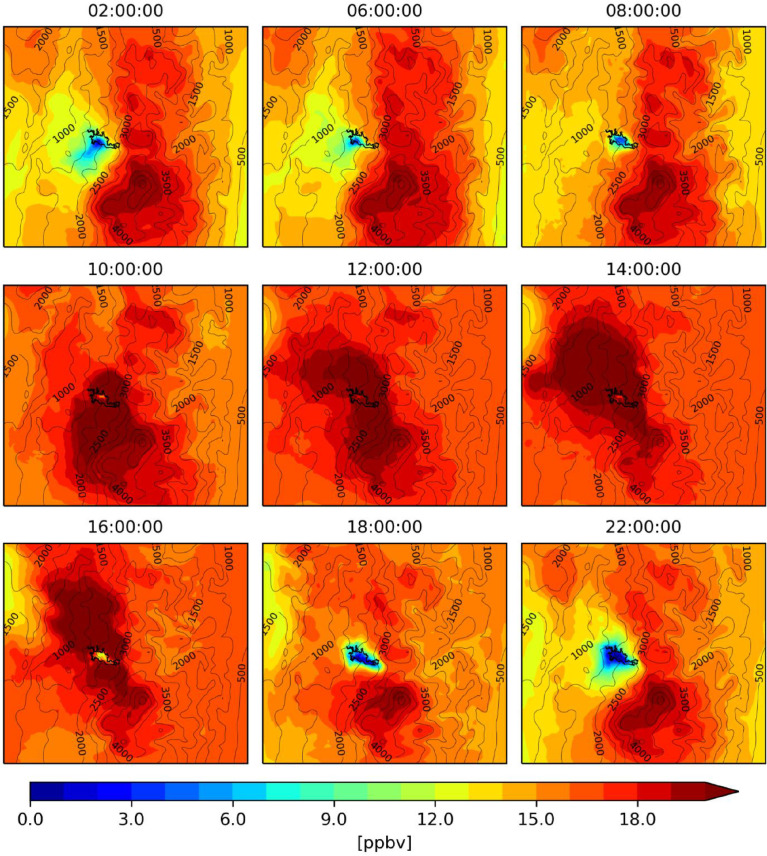
Hourly mean ozone concentrations estimated with MEGAN-WRF-Chem for the period of simulation (June 3, 00:00 UTC to July 1, 00:00 UTC 2018).

**Fig. 7:**
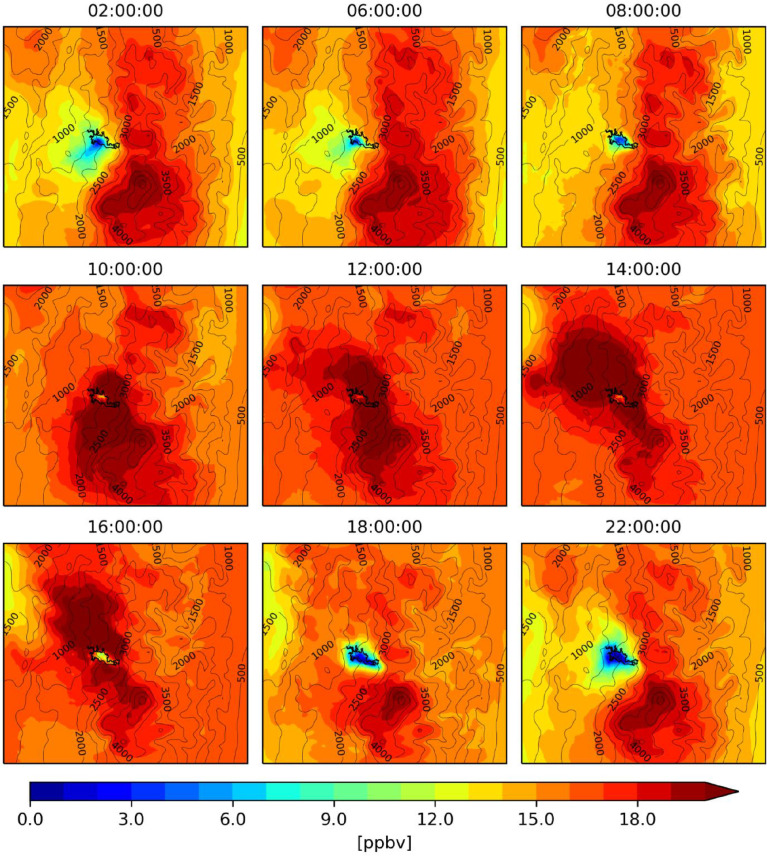
Hourly mean ozone concentrations estimated with BIGA-WRF-Chem for the period of simulation (June 3, 00:00 UTC to July 1, 00:00 UTC 2018).

The figures (Fig. 1, Fig. 2, Fig. 3, Fig. 4, Fig. 5, Fig. 6, Fig. 7) and videos (Videos 1–3) summarize the main differences in isoprene emissions according to the two estimation methods (MEGAN and BIGA), and the impact of these changes in isoprene and ozone concentration at a surface level according to the WRF-Chem modeled outputs. Finally, Table 3 presents some statistical performance metrics evaluating O_3_ forecasting accuracy with both biogenic models. The evaluation was performed against O_3_ ground measurements of concentration, with an hourly time resolution, obtained inside the urban area of the city (Lat: 5.06848, Lon: −75.51709). These data suggest that further assessment of emission factors and land use assignment in the region must be considered, in order to reduce the uncertainty of the BVOC emissions, and consequently, improve the accuracy of air quality simulations.

**Table 3:** Performance metrics for O_3_ forecasting.

	O3 [ppbv]	
Statistic	BIGA	MEGAN
MB	2.71	3.1
MGE	4.43	4.62
RMSE	5.72	5.95
NMB	0.4	0.46
NMGE	0.66	0.68
NRMSE	1.08	1.12
R	0.67	0.7

* Evaluated against ground measurements retrieve from an air quality station located in the coordinates Lat: 5.06848 and Lon: −75.51709.

## 2 Experimental Design, Materials and Methods

The WRF-Chem model version 3.7.1 was used to simulate air quality in a region of the tropical Andes (Extent: −75.8954, −74.9054, 4.6229, 5.5229) for a period of 28 days in 2018 (June 3 to July 1). The area is characterized for dense and diverse vegetation, and high climatic variability related to the drastic altitudinal changes (terrain elevations varying from 250 to more than 4000 m.a.s.l.) [2]. Average temperatures inside the simulation domain range from 28 °C in the lower elevation areas, to −3 °C in the higher mountain peaks. Two simulations were made to test the sensitivity of the model to the biogenic emission inventories (MEGAN and BIGA). Details of the inputs to the models, WRF-Chem settings, and outputs postprocessing are given below.

### 2.1 Inputs

*Initial and boundary conditions:* Meteorological data were retrieved from the National Center for Environmental Prediction (NCEP) Global Forecast System (GFS) Final Analysis (FNL) with a horizontal grid spacing of 0.25° and 6 h intervals (https://rda.ucar.edu/datasets/ds083.3/) and chemical data were obtained from the Community Atmosphere Model with Chemistry (CAM-Chem) simulations [6].

*Anthropogenic emission inventory:* Anthropogenic emissions were included in the model using a local emission inventory [5]. The inventory was disaggregated and speciated as specified in the emissions section of the study of [4]. Then, an emission file compatible with WRF-Chem was generated using the AAS4WRF emission preprocessor [7]. The final file is provided in the Mendeley repository.

*Online/Global biogenic emission inventory:* Online biogenic emissions were estimated using the MEGAN model couple with WRF-Chem. The gridded emissions were obtained with a temporal resolution of 1 h and a spatial resolution of 1 km – 1 km.

*Offline/Local biogenic emission inventory:* Offline biogenic emissions were estimated using the BIGA model [2] with a temporal resolution of 1 h and a high spatial resolution of 0.1 km – 0.1 km, in order to capture the high climatic variability and varied vegetation of the area of study, caused by the high altitudinal changes. The emissions were later aggregated to a resolution of 1 km – 1 km to be included in the WRF-Chem simulation using the AAS4WRF emissions preprocessor [7].

To execute BIGA, the following information was needed: (1) DEM downloaded from the Advanced Spaceborne Thermal Emission and Reflection Radiometer (ASTER) online repository (https://asterweb.jpl.nasa.gov). The DEM was later resample using GIS software to meet the horizontal resolution needed (0.1 – 0.1 km). (2) LCU map for the area of interest retrieved from the Colombian Environmental Information System (SIAC in Spanish) online repository (http://www.siac.gov.co/catalogo-de-mapas). The LCU map was converted from polygon to raster format using GIS software (3) Ground measurements of temperature and solar radiation were obtained from sixteen meteorological stations located in the area of interest (see Table 2) using the Caldas Environmental Data and Indicators Center (CDIAC in Spanish) online platform (http://cdiac.manizales.unal.edu.co/indicadores/public/searchClimatological). The records were average from five minutes to an hourly resolution. Missing data were filled using mean values. The prior information was made available in the Mendeley repository.

### 2.2 WRF-Chem configurations

Two air quality simulations were performed using each of the biogenic emission inventories previously describe. The model configurations used were defined according to the suggestions of Cifuentes et al. [4] and are listed in Table 4.

The WRF-Chem module named module_cbmz_addemiss.F was modified to include the local biogenic emissions through the same channel that local anthropogenic emissions are introduced into the model. The modified module was made available through the Mendeley repository, in the folder WRF-Chem_Inputs. The changes are found in lines 90, 130, 164–165 y 229 of the module. Note that WRF-Chem must be re-compiled after modifying the module.

### 2.3 Outputs postprocessing

Model outputs were obtained on an hourly time resolution for the period of simulation, leading to a time dimension of 673 records. NCO operators were used for averaging the data into hourly mean values (time dimension of 24 records) and to subset the variables of interest presented in Table 1. Then, Python software was used to generate the visualizations presented within the article.

### 2.4 Evaluation of the model performance

Model outputs of O_3_, obtained with both biogenic models, were compared against ground measurements of O_3_ concentration, to assess the accuracy of the model predictions. Ground observations with an hourly time resolution were retrieved from the only station measuring this pollutant in the Manizales, which is located inside the urban area of the city (Lat: 5.06848, Lon: −75.51709). The comparisons were made using the following statistical performance metrics: Mean Bias (MB), Mean Gross Error (MGE), Root Mean Square Error (RMSE), Normalized MB (NMB), Normalized MGE (NMGE), Normalized RMSE (NRMSE), and Pearson correlation coefficient (R).

**Table 4:** WRF-Chem settings for the air quality simulations.

Item	Option used
Simulation period	Four week (28 days) period in 2018. (2018-06-03 00:00:00 UTC to 2018-07-01 00:00:00 UTC)
Spin-up time	24 h
Vertical levels	35 from the surface to 50 hPa
Nesting option	1-way nesting
Domain configuration	D1: 25 km resolution (85 × 85 grid points) D2: 5 km resolution (97 × 94 grid points) D3: 1 km resolution (100 × 110 grid points)
Initial and boundary conditions	FNL analysis (0.5° resolution; 6 h interval) CAM-Chem global model
Static data	Topography: USGS
	Land use: MODIS (20 land use categories)

**Physical parameterizations**	

Microphysics	WSM6
Cumulus convection	Grell-Freitas
Planetary boundary layer	ACM2
Long wave radiation	RRTM
Short wave radiation	Dudhia
Surface layer	Noah-LSM
Land surface	Monin–Obukhov

**Chemistry options**	

Gas-phase chemical mechanism	CBMZ
Aerosol scheme	MOZAIC (Four bins)
Photolysis	Fast-J

## Data Availability

WRF-Chem sensitivity to biogenic emission inventories in a tropical region. Global online model (MEGAN) vs local offline model (BIGA) (Original data) (Mendeley Data).

## Ethics Statement

The work did not involve the use of human subjects, animal experiments, or data collected from social media platforms.

## CRediT authorship contribution statement

**F. Cifuentes:** Conceptualization, Methodology, Software, Visualization, Writing – original draft. **C.M. González:** Conceptualization, Validation, Supervision, Writing – review & editing. **B.H. Aristizábal:** Conceptualization, Resources, Supervision, Writing – review & editing.

## Declaration of Competing Interest

The authors have no conflict of interest to report.

## Appendix Supplementary materials

Video 1. Comparison of hourly mean isoprene emissions between MEGAN-WRF-Chem and BIGA for the period of simulation (June 3, 00:00 UTC to July 1, 00:00 UTC 2018). [Please replace by video file submitted along the manuscript. Video_1.avi] Image, image 1

Video 2. Comparison of hourly mean isoprene concentrations between MEGAN-WRF-Chem and BIGA for the period of simulation (June 3, 00:00 UTC to July 1, 00:00 UTC 2018). [Please replace by video file submitted along the manuscript. Video_2.avi] Image, image 2

Video 3. Comparison of hourly mean ozone concentrations between MEGAN-WRF-Chem and BIGA for the period of simulation (June 3, 00:00 UTC to July 1, 00:00 UTC 2018). [Please replace by video file submitted along the manuscript. Video_3.avi] Image, image 3
